# Effects of Consciousness and Consistency in Manual Control of Visual Stimulus on Reduction of the Flash-Lag Effect for Luminance Change

**DOI:** 10.3389/fpsyg.2013.00120

**Published:** 2013-03-14

**Authors:** Makoto Ichikawa, Yuko Masakura

**Affiliations:** ^1^Department of Psychology, Chiba UniversityChiba, Japan; ^2^School of Computer Science, Tokyo University of TechnologyHachioji, Tokyo, Japan

**Keywords:** active observation, subjective set, controlling of stimulus change, proprioceptive information, training

## Abstract

Four experiments investigated how observers’ consciousness about their control of stimulus change affects the visual perception associated with the illusory flash-lag effect. In previous study (Ichikawa and Masakura, 2006), we found that the flash-lag effect in motion is reduced if observers were conscious that they were controlling stimulus movements by the use of computer mouse, even if the stimulus moved automatically, independently of observer’s mouse control. In the other study (Ichikawa and Masakura, 2010a), we found that the consistent directional relationship between the observer’s mouse control and stimulus movement, which is learned in our everyday computer use, is important for the reduction of the flash-lag effect in active observation. In the present study, we examined whether the reduction of the flash-lag effect in active observation requires the observers’ consciousness about their control of stimulus change, and consistency in coupling mouse movement direction and stimulus change across trials in experiments. We used the flash-lag effect in luminance change because there is no intrinsic relationship between observer’s mouse control and luminance change in our everyday computer use. We compared the illusory flash-lag effects for automatic change of the luminance with luminance change that was controlled by the observers’ active manipulation of a computer mouse. Because the flash occurs randomly in time, observers could not anticipate when the flash was presented. Results suggest that the not only observer’s consciousness of controlling the stimulus, but also consistency in coupling mouse movement direction with stimulus change, are required for the reduction of the flash-lag effect in active observation. The basis of the reduction of the flash-lag effect in active observation is discussed.

## Introduction

When a flash is presented physically aligned with a continuously moving stimulus, the flash is perceived in a lagged position relative to the moving stimulus. This is called the flash-lag effect (Nijhawan, 1994). This illusory lag effect has been found not only for positional transition, but also for transition in other visual attributes, such as changes in luminance, shape, and randomness (Sheth et al., 2000). For instance, for the luminance flash-lag observation, a stationary disk appeared on one side of the fixation point at the start of each trial and gradually increased (or decreased) its luminance. The second disk was briefly presented for one frame on the opposite side of the fixation point. Even if the luminance of those disks was the same, the first disk looks brighter (or dimmer) than the second one. This illusion has been explained by extrapolation of the delay of the visual processing (Nijhawan, 1994), postdictive processing for the moving stimulus (Eagleman and Sejnowski, 2000), differences in the processing time between the flash and moving stimulus (Murakami, 2001), delay of shift of attention which was captured by the flash (Baldo and Klein, 1995), and so on.

Our previous study has demonstrated that a viewer’s active observation of the moving stimulus reduces the flash-lag effect (e.g., Ichikawa and Masakura, 2006). That is, the flash-lag effects in movement and luminance change were reduced if the observer actively controlled the continuous movement or luminance change of visual stimulus by the use of a computer mouse. The aim of this study is to find the necessary condition for the reduction of the flash-lag effect in active observation.

Lopez-Moliner and Linares (2006) reported that a reduction of the flash-lag effect when an observer’s key press controlled the presentation of the flash, and hence observer could predict the presentation of the flash. Other studies found that removing attention from either the flash (Murakami, 2001; Baldo et al., 2002) or the moving stimulus (Shioiri et al., 2010) increases the flash-lag effect. These findings suggest that the active observation which is associated with attention directed to either the moving stimulus or the flash may facilitate visual processing and hence reduce the flash-lag effect in active observation. However, in our previous study (Ichikawa and Masakura, 2006), even if the flash occurs randomly in time, hence cannot be anticipated, the flash-lag effect was reduced when an observer actively controlled continuous movement of visual stimulus. This result indicates that, even if observer has difficulty to attend the stimuli and flash, active observation may reduce the flash-lag effect.

Our previous study also found that, even if the stimulus moved automatically, the flash-lag effect is reduced when the observers had a consciousness (subjective mental set) that they were controlling stimulus movements (Ichikawa and Masakura, 2006). That is, when the moving stimulus was controlled by the mouse until it reached the middle point of the movement, and then it moved automatically, observers did not noticed that the stimulus movement turned to automatic. For this condition, the flash-lag effect was reduced as in the active observation although the stimulus movement was automatic when the flash was presented. From this result, one may assume that the mental set of observers that they actively control the stimulus movement may reduce the flash-lag effect.

However, subsequent findings cast doubt on the assumption that the subjective-set of control over the stimulus plays a main role for the reduction of the flash-lag effect in active observation. That is, even when observer was conscious that they control the stimulus movement, this did not reduce the flash-lag effect if the observer used an unfamiliar device to control the visual stimulus, such as trackball (Ichikawa and Masakura, 2010a) or robotic arm (Scocchia et al., 2009). In addition, we found a reduction of the flash-lag effect when upward (and downward) movement of the moving stimulus was coupled with the forward (and backward) movement of the observer’s hand, as in most computer-operating systems (e.g., MS’s Windows, Apple’s Mac OS, and Linux). However, this was not the case when the directional relationship between the stimulus movement and hand movement was reversed (Ichikawa and Masakura, 2010a). In addition, we found that even for the reversed pairing of directional relationship, the flash-lag effect was significantly reduced when observers were trained on the reversed relationship. These results indicate that learning about the everyday relationship between hand movement and stimulus transition may cause a reduction in the flash-lag effect by facilitating the visual processing through motor-sensory interaction.

The results of those previous studies do not exclude the possibility that the subjective consciousness of controlling over the stimulus has the effect to reduce the flash-lag effect if there is no factor which may disturb the visual processing. In those previous studies, the factors which are related to unfamiliarity in experimental setup (e.g., in the directional relationship between the hand movement and stimulus movement, and in the experimental devices) might disturb the visual processing. One should notice the possibility that this disturbance might cause the failure of the reduction of the flash-lag effect although observer’s subjective consciousness of controlling the stimulus may have the effect to reduce the flash-lag effect.

In the present study, we examined whether the consciousness of controlling over the stimulus may reduced the flash-lag effect when there is no factors which may disturb the visual processing. That is, we used the flash-lag effect in luminance change (Sheth et al., 2000), in which there is no obvious intrinsic or learned directional relationship between hand movement and luminance change of a stimulus in any computer-operating systems, and for which we found the reduction of the flash-lag effect in active observation (Ichikawa and Masakura, 2006). In addition, we examined how both a directional relationship between the hand movement and stimulus change, and learning about these relationship affect the facilitation of the visual perception, and consequently the extent of the flash-lag effect in active observation. We conducted four experiments to examine if and how the reduction of the flash-lag effect in luminance change depends upon the consistency of directional relationship between hand movement and stimulus change while the observers were conscious of controlling the luminance change of the stimulus. We will discuss the results of these experiments and the role of consciousness of controlling the stimulus, and consistency in the directional relationship between the hand movement and stimulus change in visual processing.

## Experiment 1

It is possible that reversing the directional relationship between hand movement and luminance changes used in the previous study (Ichikawa and Masakura, 2006) would diminish the degree to which the flash-lag effect was reductive effect by active observation. If so, then this would implicate the impact of some implicit learning of a specific directional relationship between the hand movement and luminance change due to our routine use of the computer mouse. Therefore, Experiment 1 examined whether an observer’s control of a computer mouse reduces the flash-lag effect if the directional relationship between hand movement and luminance changes was reversed relative to the relationship examined in that previous study.

### Method

#### Observers

Five observers participated in the first experiment. They were graduate or undergraduate students; their ages ranged from 21 to 28 years. Although three of them had took part in the experiment in which we examined the effects of active observation on the flash-lag effect for motion, and showed significant reduction of the flash-lag effect, they were naïve as to the purpose of this study. All of them had normal or corrected-to-normal visual acuity and were right-handed, and all had used a personal computer with a computer mouse for at least 4 years.

#### Stimuli and apparatus

**Figure 1:**
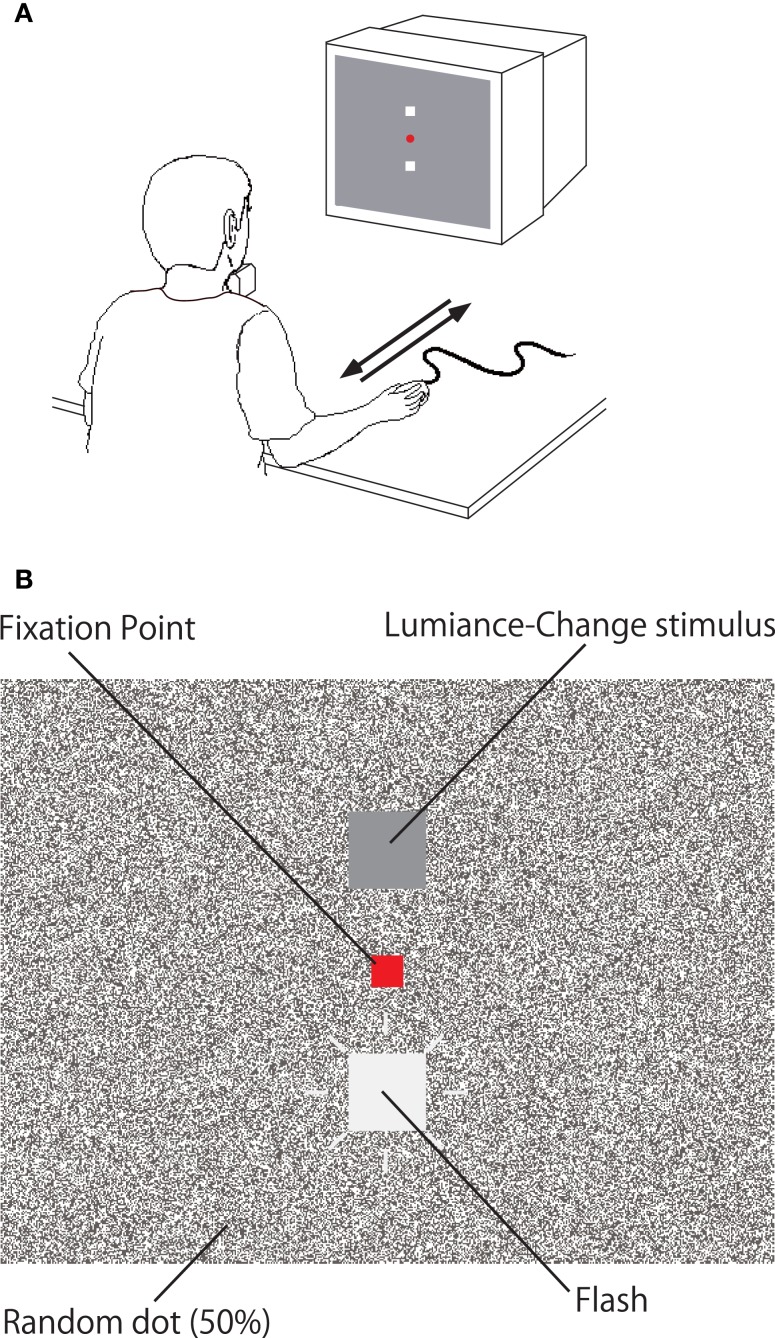
**Apparatus for Experiment 1**. **(A)** In the Manual condition, the forward (or backward) movement of the computer mouse on the desk was coupled with the decrement (or increment) of the luminance in the luminance change stimulus. **(B)** Display used in Experiment 1.

We used the same apparatus and setting as used in our previous studies (Ichikawa and Masakura, 2006, 2010a). A personal computer (Apple Macintosh G4 with Mac OS 9) presented stimuli by the use of Vision Shell programing on a 21″ display (Eizo T962, 75 Hz). The viewing distance was about 50 cm. The observer sat on a chair in front of a desk, with the head fixed on a chin rest, and grasped the computer mouse (Apple Pro Mouse M5769) with the right hand on the desk (Figure 1A). A computer keyboard (Sanwa Supply SKB-M1090H) was placed by the observer’s left hand. The mouse and keyboard were connected to the computer by USB cables.

The center of the display was at the eye level of the observer. The luminance change stimulus was a stationary square (57.3 arcmin × 56.9 arcmin) whose luminance changed from 31.1 to 81.4 cd/m^2^ (or from 81.4 to 31.1 cd/m^2^). It was presented 1.0° below or above the red fixation point (19.0 arcmin × 19.1 arcmin) that was located at the center of the display (Figure 1B). In order to handle luminance of the luminance change stimulus, we conducted Gamma correction, and choose the range of color look-up table, which enables monotonic luminance change in the stimulus. We used 50% of random dot display as background in order to reduce the afterimage of the flash. The size of a dot in the background was 2.4 arcmin × 2.4 arcmin, and the luminance of the white and black dots were respectively 85.1 and 1.0 cd/m^2^. A red horizontal line (334.3 arcmin × 2.4 arcmin) was presented at the bottom or top of the display (about 15.2° above or below the fixation point) to indicate the start position for the mouse.

During the luminance change, a flash stimulus (57.3 arcmin × 56.9 arcmin) was presented for 13.3 ms, 1.0° above or below the fixation point with random timing. There were nine conditions for the luminance of the flash stimulus (ranging from 46.9 to 65.9 cd/m^2^ by about 2.4 cd/m^2^ step).

#### Procedure

Procedures were very similar to those that we used in our previous study to investigate the effects of active observation on the flash-lag effect in luminance change (Ichikawa and Masakura, 2006). Observer’s task was to judge whether the flash was brighter than the luminance change stimulus at the moment of the flash presentation. There were two observation conditions in which the luminance change stimulus was controlled in different ways. In the first condition (the Manual condition), the luminance of the luminance change stimulus changed with the position of the computer mouse that the observers manually moved forward (away from the body) or backward (toward the body) on a desk. That is, forward and backward mouse movements were respectively coupled with the decrement and increment of the luminance in the luminance change stimulus. This directional relationship between the hand movement and luminance change was consistent throughout the session. This relationship was opposite to that used in our previous study in which we found the reduction of the flash-lag effect in luminance change (Ichikawa and Masakura, 2006). About 27.0 cm of hand movement in depth dimension on the desk corresponded to the luminance change from 31.1 to 81.4 cd/m^2^ of the luminance change stimulus.

Observers were instructed to fixate on the red fixation point and to move the mouse for about 2 s from the darkest (or brightest) to the brightest (or darkest) appearance to create continuous changes in luminance with a constant change velocity. If the luminance change took less than 1,600 ms or longer than 3,200 ms, the experimenter told the observer that the hand movement was out of the acceptable range and that he or she should move his/her hand faster or slower. That trial was presented again at the end of a block. In order to learn both the acceptable hand movement rate and that the hand movement changes the luminance of the stimulus, observers had a practice session with at least 40 trials before the experimental trials until the observer’s hand movement was within the acceptable range (from 1,600 to 3,200 ms) for at least 10 consecutive trials. In the practice session, observers moved the mouse while viewing a display that showed the luminance change stimulus with the red fixation point and index line, but no flash stimulus.

In the second condition (the Automatic condition), the luminance change stimulus changed its luminance with the constant velocity (change rate) that was determined by the average velocity for the first conditions for each individual. Therefore, sessions for this condition were conducted just after all of the sessions for the first condition. Each trial began with presentation of a fixation point and luminance change stimulus, as in the first condition. After a randomly determined time interval (1,000–2,000 ms) after the observer pressed the space key, the luminance change stimulus began to change its luminance.

There were five blocks for each of the Manual and Automatic conditions. In each block, 36 stimulus conditions [luminous lag between the stimuli (9) × direction of the stimulus movement (2) × vertical position of the luminance change stimulus (2)] were presented once in random order (Total numbers of trials were 360 for an observer). At the beginning of each trial, the red fixation point and the red horizontal line were presented. For the Manual condition, the observers located the computer mouse at the start point on the desk in accordance with the position of the horizontal red line. When the observers pressed the space key to start the trial, the luminance change stimulus was presented below or above the fixation point. In each condition, the observer’s mouse control (Manual condition) or key press (Automatic condition) started the luminance change of the stimulus. After a randomly determined time interval (0–400 ms) after the luminance change stimulus passed its luminance mid point, a flash was presented for 13 ms with one of the nine possible luminance levels. After the luminance change stimulus reached the end point of the luminance change, the observers pressed one of two keys to report whether the flash was brighter or darker than the luminance change stimulus.

In all of the experiments in this study, after all of the experimental sessions, the observers reported which of the conditions is the easiest in the luminance judgment, and guessed in which conditions their judgment was the most valid. In addition, they reported whether they felt that they controlled the luminance change of the stimulus during the sessions for each condition.

### Results and discussion

In the Manual condition, means of the time that each observer took in moving the mouse ranged from 2,313 to 2,421 ms (*M* = 2,354 ms). The mean of the velocities (change rate) in the luminance change stimulus for each observer ranged from 20.8 to 21.8 cd/m^2^/s (*M* = 21.4 cd/m^2^/s) for the Manual condition. The SD of the velocity within an individual observer ranged from 1.5 to 1.8 cd/m^2^/s (*M* = 1.6 cd/m^2^/s) for the Manual condition. These small SD indicate that the observer complied with instruction to move the mouse with a constant and stable velocity in the trials for these conditions. All of the observers reported that they controlled the luminance change in the Manual condition although they never felt that they controlled the luminance change in the Automatic condition.

**Figure 2:**
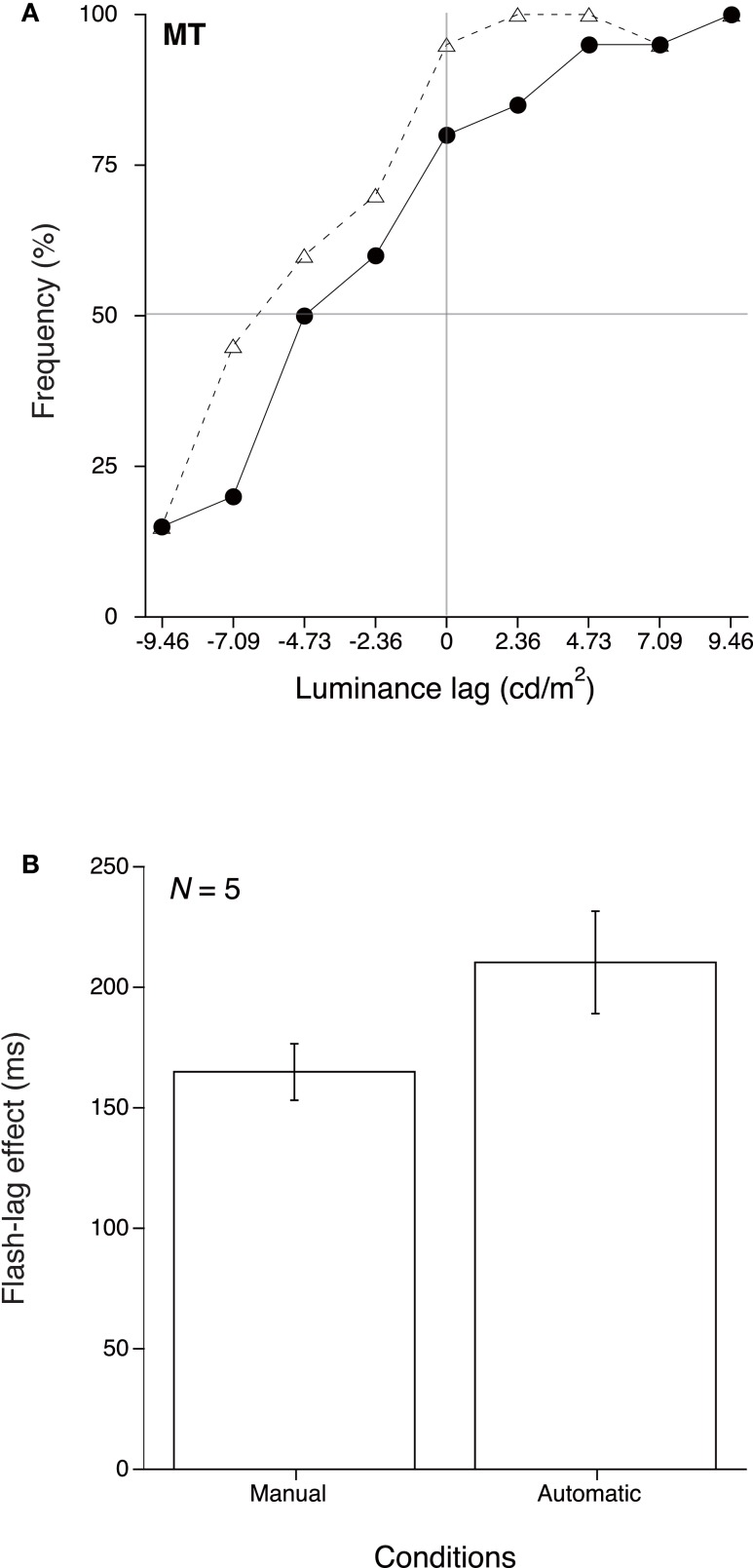
**Results of Experiment 1**. **(A)** Example of a typical observer (MT). Solid circles and open triangles represent, respectively, results of the Manual and Automatic conditions. **(B)** Mean and SE of the 50% thresholds for the two conditions.

Figure 2A shows results for a single observer, as an example. The vertical axis indicates the frequency of trials that the observer reported that the luminance change stimulus exceeded the luminance level of the flash. The horizontal axis shows the luminance lag between the luminance change stimulus and the flash. A zero point on this axis represents the luminance change stimulus and flash with the same luminance level. Therefore, on these trials, the appropriate frequency would be to judge stimulus brighter than flash 50% of the time. However, in the Manual condition, MT judged that the luminance change stimulus was brighter than the flash on about 80% of the trials and in the Automatic condition this rose to 95% of the trials.

A Probit analysis (Finney, 1971) determined as the 50% threshold for the response that the luminance change stimulus exceeded the luminance level of the flash. The flash-lag effect was derived from the division of the threshold by velocity of the luminance change for each observer. Figure 2B shows the means of the result thresholds value in each of the two conditions, averaged over the five observers. This figure reveals a clear reduction of the flash-lag effect for the Manual condition relative to the Automatic condition. A paired *t*-test, used to compare the observed means for these two conditions, indicated that the difference between the Manual and Automatic conditions was significant [*t*(4) = 3.061, *p* < 0.05]. This result indicates that coupling the forward and backward hand movement with the decrement and increment of luminance leads to a reduction of the flash-lag effect that is similar to the reduction reported in Experiment 2 in that previous study in which forward and backward hand movements were respectively coupled with the increment and decrement of luminance (Ichikawa and Masakura, 2006).

We conducted a 2 × 2 mixed design ANOVA to compare the flash-lag effects in luminance change in this study with that found in our previous study (Ichikawa and Masakura, 2006; with *N* = 6 observers) by the use of the experiment (present, or previous experiment) as a between factor, and observing condition (Manual, or Automatic) as a within factor. Only the main effect of the observing condition was significant [*F*(1, 9) = 8.633, *p* < 0.05]. Thus, we could duplicate the results of our previous study with the flash-lag effect for luminance change for the opposite directional combination between hand movement and luminance change. The present results, together with the those of the previous study, suggest that it is likely that consistency in the relationship between the hand movement and luminance change leads to reduction of the flash-lag effect for luminance change, and that the flash-lag effect is reduced in active observation regardless of whether the forward and backward hand movements were respectively coupled with the increment or decrement of the luminance.

The result that the naïve observers’ perception in the Manual condition was more valid than that in the Automatic condition is not congruent with the observers’ consciousness. That is, all of them guessed that their performance is more valid in the Automatic condition because, in the Automatic condition, they could concentrate on the visual stimuli although, in the Manual condition, they had to pay attention to the hand movement in order to move the mouse with a constant velocity. This incongruence between the measurement of the illusory flash-lag and observers’ subjective introspection suggest that observers did not aware the reduction of the flash-lag effect in the active observation.

## Experiment 2

The second experiment was designed to pursue the possibility that consistency in the directional relationship between the stimulus luminance change and the observer’s hand movement is the source of reduction in the flash-lag effect. In a previous study (Ichikawa and Masakura, 2010a), we found that the active control of stimulus movement reduced the flash-lag effect in motion only when the directional relationship between the hand movement and stimulus movement corresponded to the directional relationship in the popular computer OS. The luminance change task, however, offer the advantage of having no such intrinsic or default (routine) relationship between stimulus change and hand movements. Therefore, in the present task observers should not have acquired a learned preference for a certain directional relationship between the stimulus changes and hand movements.

In both of Experiment 1 in the present study and Experiment 2 in the previous study (Ichikawa and Masakura, 2006), observers felt that they controlled over the luminance change of the stimulus by the use of a computer mouse. In addition, in our previous studies (Ichikawa and Masakura, 2006, 2010a), observers reported that they always felt that they controlled the stimulus movement in any conditions in which they moved the stimulus by the use of computer mouse, even if the directional relationship between hand movement and stimulus motion was inconsistent. Therefore, we expected that observers would have consciousness of controlling over the luminance change of the stimulus regardless of the directional relationship between hand movements and stimulus change. Because there are no acquired biases for directional relations of action and luminance change in stimulus, one might anticipate that observer’s consciousness would reduce the flash-lag effect, regardless of the directional relationship or their consistency over trials in a session. If so, that consciousness would be a sufficient condition for reduction of the flash-lag effect regardless of the consistency of directional relationship between hand movements and luminance change of the stimuli. In the second experiment, we examined this notion.

### Method

#### Observers

Nine new observers took part in the second experiment (four females and five males). Five of them had took part in the experiment concerning with the effects of active observation on the flash-lag effect in motion, and showed significant reduction of the flash-lag effect in active observation. All of them were naïve as to the purpose of this study. In addition, one of the two authors (Makoto Ichikawa), who had taken part in the experiments in the previous study (Ichikawa and Masakura, 2006, 2010a), participated in the experiment. Ages of the observers ranged from 21 to 44 years. All had normal or corrected-to-normal visual acuity and were right-handed, and all had used a personal computer with a computer mouse for at least 4 years.

#### Stimuli and apparatus

The set up of the equipment and the stimulus configuration were the same as in Experiment 1. As in Experiment 1, there were nine conditions for the luminance of the flash stimulus (ranging from 46.9 to 65.9 cd/m^2^ by about 2.4 cd/m^2^ step).

#### Procedure

Three observation conditions controlled the luminance change stimulus in different ways. In the first condition (Forward-Increment condition), the luminance of the luminance change stimulus was yoked to the position of the computer mouse that the observers manually moved forward or backward on a desk. The forward and backward movements of the mouse were respectively coupled with the increment and decrement of the luminance in the luminance change stimulus, as in our previous study (Ichikawa and Masakura, 2006). In the second condition (Forward-Decrement condition), the directional relationship between luminance change and mouse movement was the same as that used in Experiment 1 (i.e., the reverse of the Forward-Increment condition). In these two conditions, about 27.0 cm of mouse movement in depth dimension on the desk corresponded to the luminance change from 31.1 to 81.4 cd/m^2^ of the luminance change stimulus. In this experiment, the two different mapping of mouse direction onto stimulus change (Forward-Increment, or Forward-Decrement) were presented on different trials within the same block, and thereby violated the consistency of these stimulus-mapping in each block. In all other respects, the procedure was the same as those used in the Manual condition in Experiment 1.

In the third condition (Automatic condition), the luminance change stimulus changed its luminance with the constant velocity (change rate) that was determined by the average velocity for the first and second conditions for each individual. At the beginning of each trial, the fixation point and luminance change stimulus were presented, as in the first and second conditions. After the random interval ranging from 1,000 to 2,000 ms after the observer pressed the space key, the luminance change stimulus started to change its luminance with a constant change rate. The blocks for this condition immediately followed the blocks for the first and second conditions.

There were five blocks in which both the Forward-Increment and Forward-Decrement conditions were presented. In each block of trials for these conditions, 72 stimulus conditions [directional relationship between the hand and luminance change (2) × luminous lag between the stimuli (9) × direction of the stimulus movement (2) × vertical position of the luminance change stimulus (2)] were presented in random order. There were also five blocks for the Automatic condition. In each block for the Automatic condition, 36 stimulus conditions [luminous lag between the stimuli (9) × direction of the luminance change (2) × vertical position of the luminance change stimulus (2)] were presented in random order (Total numbers of trials were 540 for each observer). Between the blocks, observers had short rests.

At the beginning of each trial, the red fixation point and the red horizontal line were presented. In the Forward-Increment and Forward-Decrement conditions, in accordance with the position of the horizontal red line, the observers located the computer mouse at the start point on the desk for the Forward-Increment and Forward-Decrement conditions. When the observers pressed the space key to start the trial, the stimulus was presented at the below or above the fixation point. In each condition, the observer’s mouse control (Forward-Increment or Forward-Decrement condition) or key press (Automatic condition) started the luminance change of the stimulus.

### Results and discussion

For all trials in the Forward-Increment and Forward-Decrement conditions, the means of the time for each observer to move the mouse ranged from 2,310 to 2,394 ms (*M* = 2,361 ms) for the Forward-Increment condition, and from 2,281 to 2,415 ms (*M* = 2,356 ms) for the Forward-Decrement condition. The mean change rate of the luminance was recorded in each trial; the mean of the change rates for each observer ranged from 21.0 to 21.8 cd/m^2^/s (*M* = 21.3 cd/m^2^/s) for the Forward-Increment condition, and from 20.9 to 22.1 cd/m^2^/s (*M* = 21.4 cd/m^2^/s) for the Forward-Decrement condition. All of the observers reported that they felt that they controlled the luminance change in the Forward-Increment and Forward-Decrement conditions although they did not in the Automatic condition.

**Figure 3:**
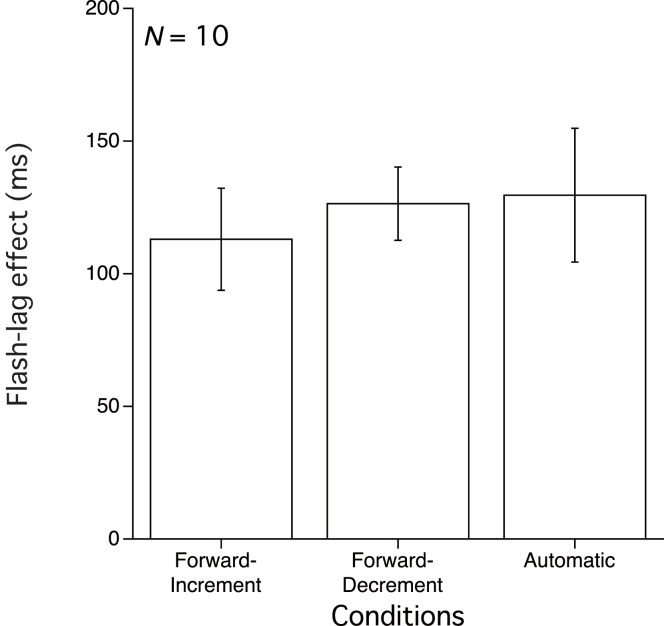
**Results of Experiment 2**. Mean and SE of the 50% threshold for the three conditions.

As in Experiment 1, the flash-lag effect was derived from the luminance lag using Probit analysis determined as the 50% threshold for the response that the moving stimulus passed the level of the flash. Figure 3 shows the means of the 50% thresholds in each condition for the 10 observers. This figure reveals no significant difference among three conditions. A one-way repeated measure ANOVA compared means of these three conditions using the data from the 10 observers (Figure 3). The main effect of condition was not significant [*F*(2, 18) = 0.527, *p* > 0.05].

All of the observers, including MI (one of the authors) reported that they felt that they controlled the luminance change in the stimulus in both the Forward-Increment and Forward-Decrement conditions. In addition, they reported that there were no subjective differences between these two conditions in difficulty in controlling the stimulus luminance and in judging the relative luminance of the flash and the luminance change stimulus. This suggests that the observers were conscious that they controlled the luminance levels through their movement of a mouse, as in Experiment 1, and in our previous study (Ichikawa and Masakura, 2006). However, that consciousness was not accompanied by a significant reduction in the flash-lag effect for these two Manual conditions relative to the Automatic condition even if there is no inconsistency based on learning in the directional relationship between hand movement and stimulus change. This result suggests that the observers’ consciousness that they control the stimulus luminance is not a sufficient condition for the reduction of the flash-lag effect in luminance change.

As in Experiment 1, the observers guessed that their performance was more valid in the Automatic condition because they could more concentrate on the luminance judgment in the Automatic condition, than in the other two conditions. However, their guess was not congruent with the obtained flash-lag effect.

## Experiment 3

Experiment 2 produced no significant reduction of the flash-lag effect based upon observers’ manual control of the stimulus luminance although such effects were evident in Experiment 1, as well as in our previous study (Ichikawa and Masakura, 2006). In Experiment 2, the Forward-Increment and Forward-Decrement conditions were conducted randomly within the same block. Therefore, it is possible that the inconsistency of the directional relationship between the hand movement and luminance change impaired the effect of active observation on the flash-lag effect.

In Experiment 3, we examined whether observer’s manual control of a computer mouse can reduce the flash-lag effect when the directional relationship between the hand movement and luminance change is consistent within each block. In Experiment 3, the seven observers who took part in the second experiment conducted the manual and Automatic conditions where the Manual condition involved only the Forward-Decrement mapping of hand movement and stimulus luminance changes, as in Experiment 1.

### Method

#### Observers

The seven naives of 10 observers who took part in Experiment 2 participated in Experiment 3 4–8 weeks after the second experiment.

#### Stimuli and apparatus

The set up of the equipment and the stimulus configuration were the same as in Experiment 1.

#### Procedure

The procedures were the same as in Experiment 1 except that the observers who served in Experiment 2 (and also experienced the opposite, and inconsistently presented directional relationship between the hand movement and luminance change) participated in this experiment. There were five blocks for each of the Manual (Forward-Decrement) and Automatic conditions. In each block, 36 stimulus conditions [luminous lag between the stimuli (9) × direction of the luminance change (2) × vertical position of the luminance change stimulus (2)] were presented in random order.

### Results and discussion

For all trials in the Forward-Decrement condition, the observers’ mean times for moving the mouse ranged from 2,342 to 2,408 ms (*M* = 2,367 ms). The mean change rate of the luminance was recorded in each trial; the mean of the change rates for each observer ranged from 20.9 to 21.5 cd/m^2^/s (*M* = 21.3 cd/m^2^/s). The SD of the change rate within an individual observer ranged from 1.3 to 1.6 cd/m^2^/s (*M* = 1.5 cd/m^2^/s) for the Manual condition. No consistent difference in the change rate was observed in the Manual condition between Experiments 1 and 3.

**Figure 4:**
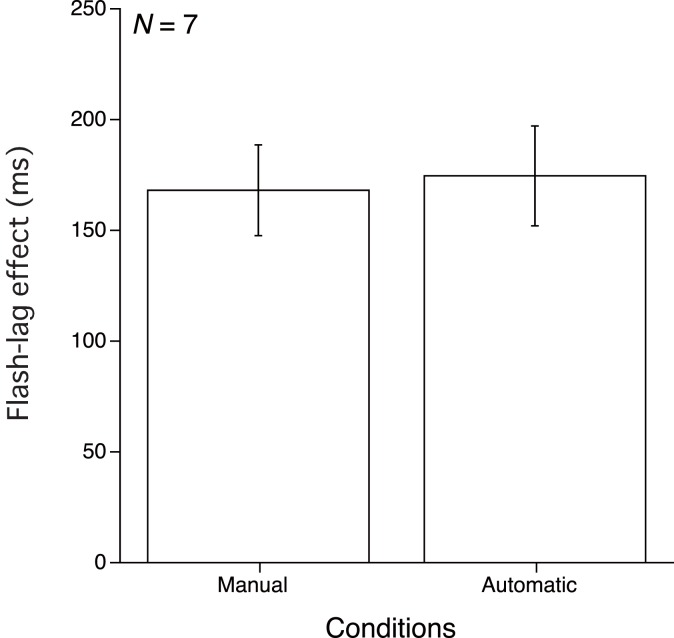
**Results of Experiment 3**. Mean and SE of the 50% threshold for the two conditions.

As in Experiments 1 and 2, the flash-lag effect was derived from the luminance lag based upon the 50% threshold for the response that the luminance change stimulus exceeded flash luminance level. Figure 4 shows the means of the 50% thresholds in each of the two conditions averaged over data from seven observers. A paired *t*-test comparing the means of these two conditions reveals no statistically significant difference between them [*t*(6) = 0.282, *p* > 0.10].

As in Experiment 1 and 2, all of the observers reported that they felt that they controlled the luminance change in the Manual (that is, Forward-Decrement) condition. Such a finding indicates that, even if the observers were conscious that they controlled the luminance change of the stimulus as in Experiment 1 (and as in our previous study Ichikawa and Masakura, 2006), and even if a consistent directional relationship between the hand movement and luminance change was maintained during the experiment, manual control of the stimulus luminance, nor the conscious of controlling over the stimulus could not reduce the flash-lag effect in the Manual condition. This result suggests long lasting effects of the prior (Experiment 2) experience of an inconsistent relationship between the hand movement and luminance change on the flash-lag effect, which stretch over several weeks.

## Experiment 4

In our previous study (Ichikawa and Masakura, 2010a), we found that the flash-lag effect in motion was significantly reduced after the training session of 360 with an unfamiliar directional relationship between the hand movements and stimulus motions on computer display. In Experiment 4, we examine whether training with a specific directional relationship between the hand movement and luminance change (Forward-Decrement condition) can reduce the flash-lag effect in a luminance change if an observer has had prior experiences with inconsistencies in the directional relationship between the hand movement and luminance changes. After the training, observers would be able to more easily control the luminance of the visual stimulus with less attention to the hand movement. This easiness might reduce the cognitive load in active observation, and consequently reduce the flash-lag effect. In order to examine whether this is the case, in Experiment 4 we used observers from Experiment 3, and Experiment 2 as well who showed no reduction of the flash-lag effect in the Forward-Decrement condition in Experiment 3.

### Method

#### Observers

The six of seven observers who took part in Experiment 3 participated in Experiment 4 from 27 to 32 weeks (about 6 months) after the third experiment.

#### Stimuli and apparatus

Equipment and stimulus configuration were the same as in Experiment 1.

#### Procedure

Experiment 4 consisted of two sessions; a training session and a post-training session. In the training sessions, procedures were similar to those of our previous study involving the flash-lag effect in motion (Ichikawa and Masakura, 2010a). That is, over 10 blocks of 36 trials each, observers were instructed to move the mouse in about 2,400 ms from the start line to the goal line. During the training sessions, the acceptable time for this hand movement in the Manual condition ranged from 2,000 to 2,800 ms; this range was narrower than that of Experiments 1 and 2. If the movement took longer than 2,800 or less than 2,000 ms, a low beeping sound notified an observer that the velocity was out of the acceptable range. In addition, if the time for the movement was within the range between 2,373 and 2,427 ms, a high beeping sound notified the observer that the movement was in the center of the acceptable range. Following the 360 training trials (10 blocks), observers had a post-training session in which they had 10 additional trial blocks. This numbers of training trials was sufficient to reduce the flash-lag effect significantly for moving stimulus in our previous study (Ichikawa and Masakura, 2010a). In the post-training session, the Manual condition (Forward-Decrement condition) was presented for five blocks followed by the Automatic condition for five blocks (procedures in both conditions were the same as those of Experiments 1 and 3).

### Results and discussion

For all of the sessions in the Manual condition, the mean amount of time that each of the six observers took in moving the mouse from the start point to the goal ranged from 2,281 to 2,500 ms (*M* = 2,394 ms) in the post-training sessions. The mean change rate of the luminance ranged from 20.1 to 22.1 cd/m^2^/s (*M* = 21.0 cd/m^2^/s). The SD of the change rate within an individual observer ranged from 1.3 to 1.7 cd/m^2^/s (*M* = 1.4 cd/m^2^/s) for the Manual condition. For the six observers, no consistent differences were observed in these values from those values in the Manual conditions of Experiments 2 and 3. All of the observers reported that they felt that they controlled the luminance change in the Manual condition although they did not in the Automatic condition both before and after the training session. They guessed that their performance in the luminance judgment was more valid in the Automatic condition. They reported there were no remarkable difference in easiness in controlling the stimulus luminance between the sessions before and after the training session while the number of the trials in which the velocity of the luminance change was outside of the acceptable range decreased from 7.1% (SD = 3.61%) to 5.4% (SD = 1.48%) in average.

**Figure 5:**
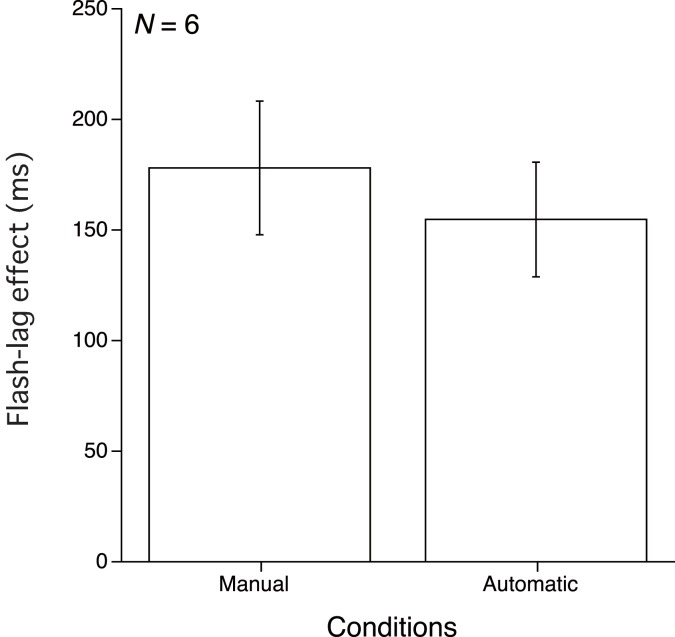
**Results of Experiment 4**. Mean and SE of the 50% threshold for the two conditions.

The flash-lag effect was derived in the same way as in the other experiments. Figure 5 shows the means of the 50% thresholds from the six observers in each condition. A paired *t*-test on mean data from the six observers found no significant difference between the Manual and Automatic conditions [*t*(5) = 1.508, *p* > 0.10]. In order to compare the flash-lag effect between before and after the training sessions, we also conducted a three by two analysis of variance in order to compare the flash-lag effects for the Forward-Decrement mapping in this study with those in Experiments 2 and 3 for the six observers who took part in all of these three experiments. The two within factors were experiment (Experiment 2, 3, or 4) and observing condition (Manual, or Automatic). We found no significant main effect [experiment factor, *F*(2, 10) = 2.795, *p* > 0.10; observing condition factor, *F*(1, 5) = 0.279, *p* > 0.10], or interaction [*F*(2, 10) = 0.779, *p* > 0.10]. These results indicate that there was no consistent variance in the flash-lag effect among these experiments.

These results suggest that, even if the observers have consciousness of controlling the stimulus during the experimental sessions, the experience of inconsistent relationship between the mouse movement and luminance change (in Experiment 2) is long lasting (for at least as much as 6 months). This inconsistency would impair the original visual facilitation process that leads to reduction of the flash-lag effect in luminance change regardless of the directional relationship between the hand movement and luminance change.

## General Discussion

Results of the present Experiment 1, as well as those from a previous study (Ichikawa and Masakura, 2006), showed that the flash-lag effect could be reduced when observers actively engage in observation of relevant stimulus, even without the learning of the directional relationship between the active hand movement and stimulus change. That is, regardless whether the forward and backward hand movements were respectively coupled with luminance increment and decrement, the flash-lag effect was reduced if the directional relationship was consistent over the trials within an experimental session. During the trials in the experiment, observers felt that they controlled the stimulus change. Together with the previous study (Ichikawa and Masakura, 2006), these results indicate that the observer’s subjective consciousness of controlling stimulus change plays important role in the reduction of the flash-lag effect in active observation when the directional relationship between the hand movement and stimulus change is consistent.

As referred in Introduction, several studies demonstrated that the prediction for the flash has effect to reduce the flash-lag effect in active observation (e.g., Baldo et al., 2002; Lopez-Moliner and Linares, 2006). In the experiments in the present study, however, because the timing of the flash was random, and therefore because the observers could not predict the timing of the flash, observer’s prediction for the flash cannot explain the reduction of the flash-lag effect in Experiment 1.

The results of the present four experiments showed that, the reduction of the flash-lag effect was restricted to the case in which the directional relationship between hand movement and stimulus change was consistent within each experiment. In those experiments, observers always felt that they controlled the luminance change of the stimulus in the active condition. The results of these experiments suggest that the reduction of the flash-lag effect in active observation require not only the consciousness of controlling the stimulus change, but also the consistency in the directional relationship between the hand movement and stimulus change to reduce the flash-lag effect. This notion is compatible with the results of our previous studies that, although the flash-lag effect was reduced in active observation for the initial relationship between hand movement and stimulus movement in direction (Ichikawa and Masakura, 2010a) and ratio in distance (Ichikawa and Masakura, 2010b), it was not reduced in the following sessions in which that relationship turned to novel ones. These results suggest the importance of the consistency in the relationship between the hand movement and stimulus change across experimental sessions for the reduction of the flash-lag effect in active observation.

We consider that the proprioceptive information, which is involved in the active observation, would be the factor which enables our visual system to reduce the flash-lag effect, in addition to the consciousness of active control of stimulus change and prediction for the timing of the stimulus presentation. There are several studies that have shown active hand movement can facilitate the visual processing of the stimuli that are coupled with observer’s own movements. For instance, active hand movement, which caused the rotation of a radial grating stimulus below the hand, enhanced the duration of the motion aftereffect for the grating stimulus if the direction of the hand movement was consistent with the direction of the visual motion (Matsumiya and Shioiri, 2008). Proprioceptive information which is related to the movement of viewing point facilitates detection of motion signal during the viewing of motion illusion figures (Spillmann et al., 2003). Moreover, tactile motion with hand would activate the human MT+ (Hagen et al., 2002; Blake et al., 2004). In short, these studies suggest that proprioceptive information of active movement of hand or body which is related to the visual motion can facilitate the processing of that visual motion. In addition, we found that the active observation reduced the reaction time both for the shape change of the moving stimulus and for the flash (Ichikawa and Masakura, 2006). This result indicates that the active observation facilitates the processing not only for the moving stimulus, but also for the area, which include both the moving stimulus and flash. This facilitation of the visual processing in terms of proprioceptive information in active observation may explain more accurate perception because the visual processing is improved by that facilitation. The results of the present study suggest that the proprioceptive information which is related to the change in stimulus may make the visual processing more accurate not only for stimulus motion, but also for luminance change in the stimulus.

Similar reduction of the flash-lag effect was found for the case in which observer attended to the moving stimulus (Shioiri et al., 2010). Because of long lasting effect of exposure to inconsistency in the directional relationship between hand movement and stimulus change, we think that the reduction of the flash-lag effect that we found in this study is caused by the proprioceptive information, rather than by the attention to the moving stimulus which is actively controlled by observer. That is, once observer is exposure to inconsistency in the directional relationship between hand movement and stimulus change is inconsistent, the visual system failed to reduce the flash-lag effect in active observation in the following experimental sessions, even several months later, and even after the hundreds of training trials with a specific directional relationship between the hand movement and stimulus change. However, as shown in Experiment 1 in the present study and our previous study (Ichikawa and Masakura, 2006), observers needed no training to acquire the reduction of the flash-lag effect if the directional relationship is consistent. These results indicate that the basis of the reduction of the flash-lag effect in active observation is established without any previous learning if the directional relationship between the proprioceptive information of the hand movement and visual information of the stimulus change is consistent, and that the inconsistency in that directional relationship impairs the basis of the reduction of the flash-lag effect in active observation for long term. Future studies should examine how the consistency in the relationship between the proprioceptive information of hand movement and visual information of stimulus change affect the flash-lag effect, and what factors may facilitate the visual processing due to active observation with specific relationship between the hand movement and stimulus change.

## Conflict of Interest Statement

The authors declare that the research was conducted in the absence of any commercial or financial relationships that could be construed as a potential conflict of interest.
